# Round about the Asylums.—IV

**Published:** 1888-04-14

**Authors:** 


### ROUND ABOUT THE ASYLUMS.—IV.

BY A MEDICAL SUPERINTENDENT.

### NOTTINGHAM BOROUGH ASYLUM.

Time, as some wiseacre remarked, flies. To most of us who are old superintendents it seems but last year since this asylum was opened, and yet here it is sending out its eighth annual report, and hard at work building additions for its ever increasing number of patients. It now contains 302, and there are 72 boarded out in other asylums. The visitors print a short abstract of the report of the Commissioners in Lunacy to the Lord Chancellor. This is, we think, a useful addition, especially to local readers. The Commissioners say that about 70 per cent. of the patients in the Nottingham Asylum are usefully employed. This is a high percentage, and may, perhaps, be partly accounted for by the fact that 82 chronic cases have been transferred to other asylums, and it is a fair presumption that Mr. Powell did not send his best working patients away—at least, if he did he acted on the Christian principle of doing unto others as he would have others to do unto him in a higher degree than the majority of superintendents with whom we are acquainted. "No restraint, and but very little seclusion, has been employed." Mr. Powell says, "It cannot be too strongly impressed upon the public that lunacy is a disease," and he thinks that some of the dislike to asylums would be lessened if they were to be known as "Hospitals for the Insane." There is much in a name, and possibly the change might help to remove old-standing prejudices; but any way it is an innovation that would have our unqualified approval. The weekly rate is given at 10*s*. 9*d*., at least that is the charge to the borough; but we nowhere found a detailed statement of the weekly cost per head, an omission which is as unusual as it is unaccountable. The recoveries were 62 per cent. on the admissions, and the deaths 8·4 on the average number resident. The former is very high. Farmers who wish to realize a competency should certainly take lessons at Leicester and Nottingham. The estate at the latter is, we believe, quite a small one, and yet it returned a profit of £295 last year.

